# *Staphylococcus aureus* in Foodborne Diseases and Alternative Intervention Strategies to Overcome Antibiotic Resistance by Using Natural Antimicrobials

**DOI:** 10.3390/microorganisms13081732

**Published:** 2025-07-24

**Authors:** Anna Phan, Sanjaya Mijar, Catherine Harvey, Debabrata Biswas

**Affiliations:** 1 Biological Sciences Program-Molecular and Cellular Biology, University of Maryland, College Park, MD 20742, USA; aphan12@umd.edu (A.P.); charvey6@umd.edu (C.H.); 2 Department of Animal and Avian Sciences, University of Maryland, College Park, MD 20742, USA; sanjmjr@gmail.com

**Keywords:** foodborne illness, *Staphylococcus aureus* food poisoning, natural antimicrobial, probiotic, prebiotic, synbiotic

## Abstract

Foodborne diseases are the most common causes of illness worldwide. Bacterial pathogens, including *Staphylococcus aureus*, are often involved in foodborne disease and pose a serious threat to human health. *S. aureus* is commonly found in humans and a variety of animal species. Staphylococcal enteric disease, specifically staphylococcal food poisoning (SFP), accounts for numerous gastrointestinal illnesses, through the contamination of food with its enterotoxins, and its major impact on human health imposes a heavy economic burden in society. Commonly, antibiotics and antimicrobials are used to treat SFP. However, a range of complications may arise with these treatments, impeding the control of *S. aureus* diseases specifically caused by methicillin-resistant *S. aureus* (MRSA). Natural alternative options to control *S. aureus* diseases, such as bacteriophages, plant-based antimicrobials, nanoparticle-based or light-based therapeutics, and probiotics, are promising in terms of overcoming these existing problems as they are environmentally friendly, abundant, unlikely to induce resistance in pathogens, cost-effective, and safe for human health. Recent findings have indicated that these alternatives may reduce the colonization and infection of major foodborne pathogens, including MRSA, which is crucial to overcome the spread of antibiotic resistance in *S. aureus*. This review focuses on the present scenario of *S. aureus* in foodborne disease, its economic importance and current interventions and, most importantly, the implications of natural antimicrobials, especially probiotics and synbiotics, as alternative antimicrobial means to combat pathogenic microorganisms particularly, *S. aureus* and MRSA.

## 1. Introduction

Foodborne diseases are considered some of the most common causes of illness, causing millions of cases and multiple outbreaks throughout the world (Heredia and García, 2018) [1]. The World Health Organization (WHO) estimates that each year, approximately 600 million people become ill and more than 400,000 people die due to unsafe food consumption and children under five years of age are at particularly high risk [2]. According to the most recent Centers for Disease Control and Prevention (CDC) Morbidity and Mortality Weekly Report (MMWR), the number of foodborne infections in 2019 increased by 15%, as compared to the previous years from 2016 to 2018, with a reported total number of infections of 25,886 in the United States (US) [3]. The CDC also reported that certain groups of people, especially adults aged 65 and older, children younger than five years, people with immunocompromised immune systems, and pregnant women, are at a higher risk for developing more serious symptoms from foodborne diseases [4]. Furthermore, the consumption of contaminated foods can cause more than 200 diseases, ranging from diarrhea to cancers [5]. The majority of foodborne illness are caused by enteric pathogenic microorganisms, including bacterial pathogens, viruses and parasites [6]. Despite advances in food safety, the current growing trend of antibiotic-resistant bacterial pathogens and limitations of traditional antimicrobial interventions are a global concern. To reduce antibiotic use or dependency, alternative strategies to tackle antibiotic resistance, including biofilm disruption, nanotechnology, alternative therapies, and rapid detection tools, are now the major priority for the biomedical researchers [7]. Following the available studies, the authors aim to focus on *Staphylococcus aureus* as a major foodborne pathogen, examining its sources, transmission vehicles, and public health impact. More importantly, we highlight recent advances in the use of natural antimicrobials as alternative intervention strategies against *S. aureus*, addressing the need for a better understanding of the mechanisms of these methods and insights on their use in combating antibiotic resistance in the food supply. By synthesizing current literature and identifying research gaps, this review aims to inform future directions in controlling *S. aureus* in different stages of food production and processing, but it also aims to highlight potential changes to food safety protocols and antimicrobial methods.

## 2. Foodborne Diseases and Food Poisoning Pathogens

Pathogens, such as bacteria, viruses, fungi, parasites, and protozoa, account for a large part of food contamination. Some of these pathogens include, but are not limited to, *Salmonella*, *Escherichia coli*, *Campylobacter*, and *Staphylococcus*, and they can produce the toxins responsible for food poisoning [5,8]. According to the CDC, the top 5 causative agents of foodborne illnesses in the US are listed as: *Norovirus*, *Salmonella*, *Clostridium perfringens*, *Campylobacter*, *and S. aureus* [9]. Although *Norovirus* is one of the top causative foodborne pathogens, foodborne bacterial pathogens are becoming a global issue due to the globalization of the food market and change in human consumption [10].

### 2.1. Major Bacterial Pathogens Responsible for Food Poisoning and Illness

Enteric bacterial pathogens are the most common causative agents of foodborne illness, and they have a variety of different properties that can lead to illness. Some of them can secrete heat-resistant toxins that can last for long periods of time on food, and a few other bacterial pathogens are spore-forming, which help them resist heat and some methods of food processing [11]. Many of them are also able to form biofilms, such as *S. aureus*, and are resistant to common detergents [10]. Many food-borne pathogenic bacteria are Gram-negative bacteria like *Salmonella enterica* serovar Typhimurium (*S. Typhimurium*), *E. coli*, and *Pseudomonas aeruginosa*, while other food-borne pathogenic bacteria are Gram-positive, including *S. aureus* and *Bacillus cereus* [11]. Some pathogens, such as *Clostridium botulinum*, *Listeria monocytogenes*, and *Vibrio*, have a lower infection rate, but are recognized to cause hospitalizations from infections [12,13,14]. Of the pathogens identified to cause foodborne diseases, *S. aureus*, *Salmonella*, *Campylobacter*, *L. monocytogenes*, and *E. coli* are among the most common, with *S. aureus* earning public attention due to its association with multidrug resistance and transmission rate between humans and animals [10].

### 2.2. Potential Foods Associated with S. aureus Foodborne Disease and Crisis with MRSA

*S. aureus* is a Gram-positive, coagulase-positive bacterium that normally colonizes the skin, nares, and pharyngeal surfaces of approximately 20–30% of humans and a variety of animal species. They can also colonize inside the body and can be isolated from the mouth, blood, mammary glands, and the upper respiratory, genitourinary, and intestinal tracts [15]. *S. aureus* secretes heat-stable staphylococcal enterotoxins (SE), which can lead to staphylococcal food poisoning (SFP). SEs can penetrate the gut lining of the gastrointestinal tract and trigger an inflammatory immune response that can cause damage to the gastrointestinal tract, and it can block the absorption of water and electrolytes, causing dehydration from diarrhea [16]. Thus, the virulence of *S. aureus* as a foodborne pathogen and the severity of infection depends on the number of SEs secreted. In animals, SEs can cause infections in food animals, such as mastitis in dairy cattle and goats and bumblefoot in poultry, which can potentially be ingested by humans [16,17]. Presently, *S. aureus*, most notably MRSA, has been detected in both animal and plant products, causing great concern due to its spread in healthcare settings [17]. MRSA has been isolated from diverse food sources, including raw meat, such as pork, lamb, chicken, and turkey, and dairy products, such as milk and cheese [18,19].

The foods associated with SFP vary significantly between regions and eating habits [20]. For instance, in the US and the United Kingdom, meat products such as ham, poultry, and beef contribute significantly to SFP outbreaks, followed by salads, pasta dishes, sandwiches, cream-filled pastries and ice cream [21,22]. On the other hand, in France, pasta salad, baked salmon, cheeses made with raw milk, mustard, and soft cheeses share a major portion in the SFP outbreaks, rather than meat products [22]. These examples highlight the diverse environments that *S. aureus* and MRSA can be isolated from, indicating its major role in food contamination and foodborne infections.

**Figure 1:**
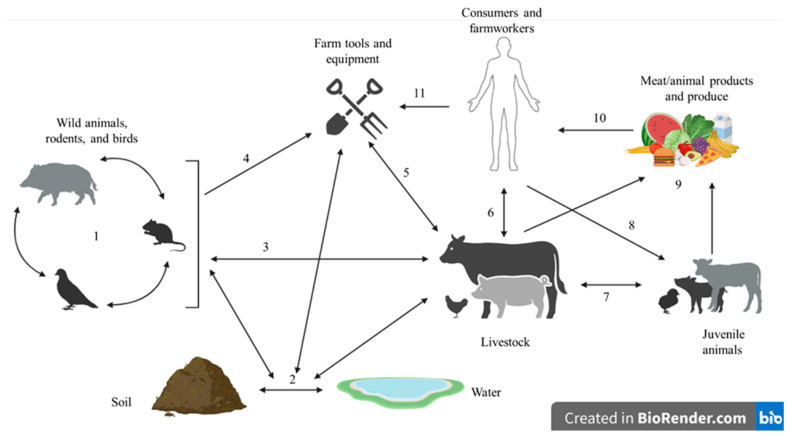
The transmission cycle of S. aureus on farms. 1: Spread in wildlife by direct contact; 2: transmission between animals and tools with the environment; 3: transmission between livestock and wildlife; 4: transmission to farm tools by contact; 5: transmission between livestock and farm tools; 6: transmission between humans and livestock; 7: spread in livestock and juvenile livestock through direct contact and vertical transmission; 8: transmission from humans to juvenile animals; 9–10: spread to humans via food products; 11: spread to tools from human handling.

A major factor leading to these infections is improper food handling practices in food industries. More than 50% of global foodborne outbreaks are associated with cross-contamination due to improper food handling or poor hygiene with food processing equipment [23,24]. *S. aureus* is commonly found on the skin and mucosal surfaces of humans and various animals, which poses a high risk of transmission through both indirect and direct contact to the soft tissue [25], as shown in Figure 1. *S. aureus* can survive in dry conditions outside of a host, including on dust and in the air, for long periods of time [26], which can increase the potential and risk of cross-contamination.

### 2.3. Incidence, Prevalence, and Food Disease Associated with S. aureus

*S. aureus* is a leading cause of healthcare-associated infections globally [10]. The European Union reported that SEs had caused around 8% of foodborne illnesses in 2016 [27], and other countries reported outbreaks of foodborne illness caused by *S. aureus* [28,29]. In the US in 2017, over 119,000 people were infected with *S. aureus*, and nearly 20,000 people died from bloodstream staphylococcal infections [30]. However, the incidence rate of *S. aureus* foodborne illness may be underreported due to a lack of systematic sample collection and laboratory diagnosis [31]. Despite the lack of a systematic method of diagnosis, there are routine testing of SEs [32], and laboratory testing is available for contaminated food [33]. However, *S. aureus* is widely prevalent across animals, as it has a broad host range.

The frequency of MRSA in food of animal origin varies by region and animal species. The frequency of MRSA was around 16.67% in raw meat and 5.71% in milk in Italy [34], which is similar to Switzerland [35]. In Germany, there was a similar frequency (1.7–9.7%) of MRSA in milk, but it was significantly higher (37.2%) in meat products [36,37]. The prevalence of MRSA was lower in the US (Iowa) and South Korea, with a frequency of 4.5% and 0.7% in raw meat, respectively [38,39]. Its broad host range, especially in food-producing animals, plays a major role in food contamination and transmission to humans [10].

### 2.4. Economic Burden of Disease or Infections Associated with S. aureus

The detrimental effects of foodborne diseases result in a great economic burden. The 2018 World Bank report estimated this burden to cost $95.2 billion in productivity loss and $15 billion in treatment costs annually in low-and middle-income countries [5]. In the US, the CDC estimates that the economic burden of foodborne illnesses is around $75 billion [40]. *S. aureus* infections are separated into methicillin-susceptible *S. aureus* (MSSA) or MRSA, with the latter being associated with increased antibiotic exposure and prolonged hospital stays [41,42]. A study conducted by Zhen et al. [43] found that MRSA is associated with higher hospital costs ranging from $3220–$9606 in addition as compared to MSSA, and a cost analysis performed by Antonanzas et al. [44] estimated that it costed up to three times as much to treat MRSA compared to MSSA. Community acquired-MRSA (CA-MRSA) is a strain of MRSA that can spread readily outside of healthcare settings in people with no history of MRSA infection, hospital admission, or indwelling devices [30]. In 2017, the total healthcare cost for treating MRSA infections was estimated at $1.2 billion for CA-MRSA and $580 million for hospital-acquired MRSA [45]. However, CA-MRSA average around $15,994 per case, a much lower cost compared to hospital-acquired MRSA at $30,998 per case, due to its higher prevalence within hospitals [46]. The substantial economic and healthcare costs associated with MRSA infections highlight the need for effective prevention and treatment strategies to reduce their impact on both individuals and society as a whole.

## 3. Occurrence of Antibiotic Resistance in *S. aureus*

In the case of staphylococcal infections, antibiotics are usually prescribed, especially against *S. aureus* due to its ability to cause a variety of infections. However, antibiotic resistance in *S. aureus* is a rising issue, as many isolates of *S. aureus* are multidrug-resistant, and it has contributed to the increasing medical costs [47,48]. Many factors play a role in the increase in resistance, most notably mobile genetic elements among various *Staphylococcus* spp. and the misuse of antibiotics [48,49,50].

### 3.1. Common Antibiotics Used Against S. aureus and the Emergence of Resistance

The beta-lactam antibiotic penicillin was first introduced as a treatment option for *S. aureus* infections in the 1940s. However, within a few years, penicillin resistance had increased by 50%, and other antibiotics, such as chloramphenicol, erythromycin, streptomycin, tetracycline, and other beta-lactam antibiotics, had to be used. *S. aureus* quickly gained resistance to these other antibiotics, and it was found that these resistances were spread through mobile genetic elements such as plasmids and transposons [50]. The synthetic antibiotic methicillin was then developed to treat *S. aureus* infections, but resistance through the *mecA* gene was found in a methicillin-resistant strain within a year. *mecA* is located on the mobile element Staphylococcal cassette chromosome *mec* (SCC*mec*), which has multiple transposon and plasmid insertion sites [37]. These insertion sites are correlated with the occurrence of multidrug resistance, especially with cross-resistance in non-beta lactam antibiotics, and they contribute to the high diversity of SCC*mec*. In 2011, a survey conducted by the European Union in raw poultry meat, the variant CC398 makes up 37.2% of MRSA, and this variant contains SCCmec elements and is most common among MRSA infected food-producing animals [37]. So far, there have been 13 SCC*mec* types reported, and several types can be found in combination within a single strain. It has been hypothesized that *S. aureus* had gained SCC*mec* via horizontal transfer from other methicillin-resistant *Staphylococcus* species, as the distribution of SCC*mec* is more diverse in other species. This indicates that they may be a reservoir of resistance that can be transferred to *S. aureus* [51].

The antibiotic vancomycin was then used to treat severe MRSA infections, as methicillin-resistant strains express the enzyme beta-lactamase, which hydrolyzes and inactivates the beta-lactam ring, rendering beta-lactam antibiotics like penicillin ineffective [52]. Vancomycin had activity against Gram-positive bacteria, and it inhibited cell wall synthesis by blocking the incorporation of the precursor lipid II to the growing peptidoglycan chain, leading to decomposition of the cell wall and subsequent cell lysis [53]. However, around the time vancomycin started being used to treat MRSA, vancomycin-resistant *Enterococci* emerged in healthcare facilities, and the first vancomycin-resistant *S. aureus* strain was isolated in a patient in 2002 [54]. Vancomycin resistance in *S. aureus* most likely resulted from the transfer of a plasmid containing the *vanA* operon from a vancomycin-resistant strain of *Enterococcus faecalis*, and it has been shown that vancomycin-resistant *S. aureus* is ecologically fit even with the addition of the *vanA* operon, suggesting that spread of the plasmid containing this operon will increase [55].

Linezolid is an oxazolidinone, a type of synthetic antibiotic, approved for use in 2000 by the FDA, and it has been used to treat various nosocomial and community-acquired infections [56,57]. Linezolid acts against most Gram-positive pathogens in humans, and it exhibits bacteriostatic activity by interfering with microbial protein synthesis by binding to the 23S rRNA of the 50S subunit. Linezolid is often used as a last resort against vancomycin-resistant MRSA, as it unaffected bacterial rRNA methylases and it can prevent the synthesis of various staphylococcal virulence factors [58]. In 2001, the first clinical strain of linezolid-resistant *S. aureus* was reported, and it was found that this isolate contained 5 copies of the 23S rRNA gene with the same mutation in domain V [59,60]. A positive correlation between the number of mutated copies of the gene and dosage of linezolid was found, suggesting that the number of mutated genes is dependent on the duration and dosage of linezolid treatment [61,62]. However, a significant increase in the rate of resistance found in *S. aureus* was through the chloramphenicol-florfenicol resistance (*cfr*) gene via both vertical and horizontal transfer. The *cfr* gene encoded a methyltransferase that targets the 23S rRNA, preventing the binding of linezolid, and it was first detected in staphylococcal isolates from animals [63]. It was reported in China that *cfr* was widespread in *Staphylococcus* isolated from animals [64], and recent studies have shown that *cfr* has also been detected in humans at varying levels, which may be due to the pressure caused by misuse of antibiotics in both the agricultural and veterinary settings [65,66].

### 3.2. Antibiotic Residues in Food and Agriculture

Antibiotic resistance in agriculture is a great public health concern, as resistant pathogens associated with livestock and produce can be easily transmitted to humans through food consumption as well as contact with contamination in the environment due to animal waste [67,68]. For example, livestock have been shown to serve as reservoirs for foodborne pathogens, such as *S. aureus*, which can be transmitted via human-livestock interactions [69]. However, there are challenges to addressing the use of antibiotics in agriculture and food production, as there is a lack of global regulation [70].

Antibiotics have been extensively used worldwide in the food industry for promoting growth and treatment of livestock and/or preserving the shelf life of food products. Mulchandani et al. [71] demonstrated that the use of antibiotics in food animals in 2020 was the highest in China, Brazil, India, US, and Australia; these 5 countries made up 58% of global antibiotic usage, and they are projected to continue being the highest users of antibiotics. Antibiotics are found to be effective in the treatment against a variety of pathogens, and they are often used to individually treat sick animals or treat an exposed group to lessen the number of sick or diseased animals [72]. In addition, antibiotics can also be administered to multiple animals simultaneously by adding them to feed or water to promote faster growth [73]. This becomes a major problem when animal feces containing antibiotics and/or their metabolites are used as fertilizer. This allows antibiotics used for livestock to be released into the growing produce, causing detrimental effects on human health upon consumption [74,75].

The most common antibiotics used in food producing animals are sulfonamides, tetracyclines, β-lactams, aminoglycosides, lincosamides, macrolides and pleuromutilins [76]. The antibiotics and their metabolites are deposited in animal tissue and/or secreted into milk for a certain period as residues. The risk of antibiotic residues is particularly greater in developing countries due to the differences in antibiotic usage in livestock and the lack of strict regulations to control, monitor, and implement the usage of antibiotics in livestock [77]. The consumption of animal products that contain drug residues beyond the maximum residue limit (MRL) can lead to serious health impacts, including cancer. Studies have shown that the consumption of animal products containing antibiotics can lead to gastrointestinal disorders, hypersensitivity reactions, and development of antibiotic resistance in bacteria, which can reduce the effectiveness of human antibiotic therapies [78,79,80]. However, despite regulations in place in some countries, the abuse and misuse of antibiotics is still widespread in the agricultural sector, and it is a major contributor to the development of resistant bacteria [70,81,82]. The CDC has highlighted antibiotic resistance as one of the most pressing problems in the world due to its rapid increase in the last decade [30], and it is estimated that more than 2.8 million antibiotic-resistant infections cause more than 35,000 deaths each year in the US [83].

Bacteria develop antibiotic resistance by several mechanisms, including bacterial cell interactions, horizontal gene transfer, and mutations in the existing bacterial genome [84,85]. Bacteria in the human microbiome can also acquire antibiotic resistance genes from nearby pathogenic species through horizontal transfer in the gut [86]. Antibiotic resistant bacteria and resistance genes can also be acquired from food of plant origins, and foods contaminated with antibiotic resistant bacteria from environment, specifically processing environment, and genes pose a serious threat of transmission and infection to the food handlers as well as consumers. Antibiotic resistance genes can be transferred to human bacterial pathogen, and these gene transfers can occur between diverse bacteria in naturally occurring environments [86,87]. Additionally, antibiotic-resistant bacteria pose a high risk to people who are constantly exposed to antibiotic residues present in their food [74]. The CDC has reported that common foodborne bacteria, such as *Salmonella*, *E. coli*, *S. aureus*, *Shigella*, and *Campylobacter*, that are derived from plant have many antibiotic-resistant strains in addition to and animal origins [83], and these increase the difficulty in treating foodborne diseases. MRSA in particular is a significant issue arising in the modern world through antibiotic resistance, and, in the last few decades, MRSA has become prevalent and widespread throughout healthcare facilities and the community. In the US, it is estimated that around 323,700 cases of MRSA patients were hospitalized, with 10,600 deaths in 2017 [86]. MRSA has also been shown to display multidrug resistance, and infections caused by multidrug-resistant MRSA often lead to prolonged hospitalizations and increased deaths [45,87]. Decreasing the usage of antibiotics is necessary to restrain the level of potential threat of antibiotic resistance in animals, humans, and whole food production. Alternative strategies to the use of antibiotics to inhibit foodborne pathogens require immediate attention in the hopes of mitigating the spread of antibiotic resistance.

## 4. Alternative Interventions to Control Food Poisoning with *S. aureus*

With the rise of multidrug-resistant S. aureus, treatment has become increasingly difficult. The introduction of new antibiotic treatments is often followed by reports of resistance, so there has been a recent push to identify and develop alternative treatment strategies to conventional antibiotics. Several approaches, such as bacteriophages, antimicrobial peptides, and plant derived compounds have been developed and shown to have potential activity against bacteria [88,89].

### 4.1. Bacteriophages

Bacteriophages (phages) are viruses that infect and replicate exclusively in bacteria. Different phages are species-specific, usually infecting only one bacterial species or even specific strains [90], providing a potential alternative treatment to various bacterial infections. Since the discovery of phages in the early 1900s, they have been actively utilized for treatment purposes [91]. For example, there have been records from as early as 1919 that show the use of phages to treat pathogens such as *Shigella dysenteriae* [92]. Phages can be split into 2 categories based on their life cycles: the virulent phage and the temperate phage [93]. The virulent phage goes through a lytic life cycle in which they use a susceptible bacterial cell to replicate and assemble new phages, leading to lysis of the host cell. The use of lytic phages presents a promising method of biotherapy, as it can be used to treat even antibiotic-resistant bacteria [94]. Temperate, or lysogenic, phages are integrated into the host genome where they exist as a prophage and leave the host intact. The host cell continues replicating as normal with the prophage indefinitely, but excision of the prophage from the genome can induce a switch to the lytic phase [94]. Temperate phages can also be used for therapy, but the integration of phage DNA into the host genome may increase the probability of transferring virulence genes from pathogenic host bacteria into resident non-pathogenic bacteria of the patient, adding a limitation on the use of temperate phage [93,95]. The USDA-Food Inspection Services (FSIS) has approved the use of multiple phages during food production [96], from proper decontamination of livestock and insurance of cleanliness of necessary equipment to the use of natural substances to extend shelf life of food products.

There are several advantages to using phages for the prevention and/or elimination of pathogenic foodborne bacteria. Whole phage and phage endolysins are found to be highly specific, abundantly available, cost effective, bactericidal, harmless to humans and animals, and non-resistant [97]. With an estimated 10^31^ phages distributed around the globe [98], they can be used constantly for the elimination of pathogenic bacteria. While there are limited clinical trials that compare the safety of high-dose versus low-dose phage therapy, an older study by Bruttin and Brussow [99] showed no adverse effects in volunteers administered low (9.0 × 10^5^ PFU/mL) and high (9.0 × 10^7^ PFU/mL) doses of *E. coli* phage T4. Many phages also have a high specificity for their host, which reduces the risk of damage to the natural microbiome in humans and animals and obviates the harsh side effects unlike antibiotics. Since the data indicates that phages do not affect eukaryotic cells, the idea of using phages for human applications is promising [100,101,102].

**Figure 2:**
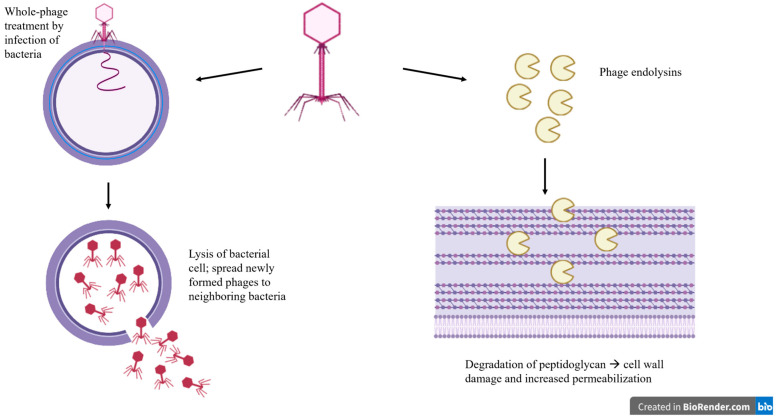
Two different methods of how phages can be used as an antimicrobial. Treatment can be performed using whole phages, resulting in infection and subsequent lysis of bacteria. Phage endolysins can be directly used on bacteria, resulting in cell wall damage.

Phages and their derivatives such as endolysins can be successfully implemented in food production and can be used as a natural antibacterial (Figure 2). In 2020, Ishaq et al. [103] conducted a study to use phages as a method of controlling *L. monocytogenes* growth on raw meat. They found that when used as a treatment, the addition of phages resulted in a 2.3-log reduction in *L. monocytogenes* over a storage period of 15 days, and phage treatment of beef did not adversely affect the color or pH of the beef samples. Other studies demonstrated that phage treatments can target specific foodborne pathogens, such as *Cronobacter*, *Salmonella*, and *B. cereus*, reinforcing the promise of using phages in food applications [104,105,106].

Due to their restricted range of action and host specificity, studies have shown phages as a promising treatment against various *S. aureus* infections. Shetru et al. [107] conducted a study in which the phage ΦDMSA-2 was used to treat a *S. aureus* skin wound model in mice. The phage-treated group exhibited faster wound healing along with a decrease in bacterial load, with a 100% survival of animals while the infected control group had a 0% survival at the end of the trial. This suggests that the phage was able to eradicate *S. aureus* and provided a protective effect to the mice, emphasizing the potential of phage treatment against MRSA. Another in vivo study by Prazak et al. [108] also demonstrated the antibacterial activity of phages against MRSA. In a mouse ventilator-associated pneumonia model, they found that the administration of aerosolized or intravenous phages rescued 50% of severely infected mice, and the combination of both administration methods rescued 91% of infected mice. These studies demonstrate that phages can be used in a wide variety of MRSA infections, suggesting their strong potential in human applications. In addition to whole phage therapy, the antibacterial activity of purified phage endolysins has also been studied against *S. aureus*. Compared to whole phages, their purified endolysins have many advantages, including a wider host spectrum, low bacterial resistance, and non-proliferation [109,110]. Studies have demonstrated the efficacy of using endolysins against MRSA in both in vitro and in vivo models. The endolysin LysP108 was reported to significantly reduce *S. aureus* concentration in vitro by damaging the outer membrane, and it was also shown to have a synergistic effect with vancomycin when used to treat MRSA-infected mice, significantly reducing bacterial load when compared with monotherapy [109]. Another recent study by Liu et al. [111] demonstrated that the endolysin LysSYL provided broad-spectrum activity, targeting multiple staphylococcal strains. They also showed that LysSYL had very strong activity against MRSA, completely lysing it within 10 min and providing protection from a lethal dose of *S. aureus* in a mouse model.

Recent studies have shown that phages have also become a promising alternative in controlling *S. aureus* in the food industry. Ngassam-Tchamba et al. [112] showed that phages had strong lytic activity against MSSA and MRSA isolated from milk in both an in vitro and in vivo mastitis model, demonstrating that phages can be used to treat bovine mastitis, improving both animal health and milk quality. Other studies also showed the antimicrobial activity of lytic phages against *S. aureus* isolated from dairy. Wen et al. [113] demonstrated that the lytic phage SapYZU15 was able to inhibit the growth of multidrug-resistant *S. aureus* isolated from milk and fresh pork, and Kwak et al. [114] also demonstrated the strong lytic activity of KMSP1 against *S. aureus* isolated from milk and cheese.

These studies show that phages are a strong contender as an alternative method to reduce *S. aureus* contamination at both the pre-harvest and post-harvest levels, lowering the risk of disease towards consumers. Their host-specificity provides the benefit of directly targeting *S. aureus* without disrupting the surround microbiome or even food quality, and studies have shown that both whole-phage or their endolysins demonstrate strong activity against *S. aureus* in clinical, veterinary, or food-related settings, demonstrating their wide range of use. The described in vivo models have also shown their efficacy in animals while also demonstrating little adverse effects, making phages a safe and sustainable alternative to traditional antibiotics in food safety and production.

### 4.2. Plant-Derived Antimicrobials

Natural antimicrobials derived from plants have been used as an alternative to inhibit or kill pathogens that cause food poisoning, and they have also been proven to have numerous health benefits (Table 1). Plant secondary metabolites are categorized into three major groups: terpenes and terpenoids, flavonoids and phenolic compounds, and N-containing compounds [115]. Over 3000 essential oils (EOs) containing these major groups have been identified [116] with the most common group being terpenes and terpenoids [117]. These EOs have been recognized for their antibacterial, antiviral, antifungal, insecticidal, and antioxidant properties, and they are also generally recognized as safe (GRAS) and impose no threat to human and animal health [118].

The antimicrobial properties of EOs and their compounds have been studied and evaluated in vitro against various pathogens [142,143,144,145]. Several researchers have proposed an inhibitory mechanism of EOs against pathogens via penetration through the bacterial membrane and changing cellular permeability and integrity, which is associated with disturbing potassium ion efflux and the leaking of internal cell components [146,147,148,149]. These insights lay the groundwork for a closer examination of specific essential oils and their unique antimicrobial effects against various pathogens.

Orange, or citrus, oil is one of the most widely used EOs because it can be cheaply extracted, and it has the potential to inhibit several foodborne pathogens, making it very applicable in the food industry [150]. Major components of orange oil that display antimicrobial properties are linalool, decanal, valencene, dodecanal, citral, geranial, citronellal, limonene, etc. [121,151,152]. In general, the antimicrobial effect of citrus EOs and their components against foodborne pathogens, such as *L. monocytogenes*, *E. coli* O157:H7, *S. aureus*, *B. cereus* [120,121], *Salmonella* [123,124,125], and *C. jejuni* [121,122], has been studied in vitro and/or in food systems. Muthaiyan et al. [119] demonstrated in vitro that exposure of *S. aureus* to orange oil resulted in the downregulation of cell wall synthesis-related genes, suggesting that it can induce cell wall damage and lysis. A recent study by Song et al. [153] confirmed this finding by visually demonstrating the inhibitory mechanism of a citrus oil against *S. aureus*. They observed via scanning electron microscopy that citrus oil damages the cell membrane, inhibiting growth in an in vitro food model. Other studies report that due to the mechanistic action of citrus oil on bacterial cell membranes, it also has an antibiofilm action against *S. aureus*, which shows its promise as a potential treatment against bovine mastitis and as a natural food preservative [121,154,155]. Ambrosio et al. [123] has also shown that orange oil may be a potentially safe alternative for treatment, as it has a selective antibacterial activity, with significantly more activity against pathogenic bacteria compared to beneficial gut bacteria.

In addition to orange oil, leaf extracts, in particular olive leaf extract (OLE), have antimicrobial properties. Studies have shown that olive leaf extract had strong antimicrobial activity against a variety of foodborne pathogens, such as *L. monocytogenes*, *E. coli* O157:H7, *S. aureus*, *C. jejuni*, *Helicobacter pylori*, and *Salmonella enterica* *serovar Enteritidis* (*S. Enteritidis*) [126,127]. The antibacterial activity of OLE is due to a variety of phenolic compounds, such as caffeic acid, rutoside, verbascoside, apigenin 7-O-glucoside, luteolin 7-O-glucoside, and luteolin 4′-*O*-glucoside [156]. OLE can also be used in combination with other natural antimicrobials to combat bacterial pathogens in a synergistic manner. For example, the combination of various polyphenols from OLE, green propolis, and *Tabebuia avellanedae* bark demonstrated strong antimicrobial activity against *S. aureus*, with an MIC as low as 0.78 mg/mL, compared to individual extracts [128]. Another study by Al-Rimawi et al. [129] showed that the combination of oleuropein, an active component of OLE, and thyme oil had strong antimicrobial activity against *S. aureus* while also lowering the concentration needed of both compounds.

Another natural antimicrobial, curcumin, has also been demonstrated to have properties against bacteria, fungi, viruses, protozoa, and parasites [157,158,159]. Its antimicrobial activity was first shown against *Mycobacterium tuberculosis* and *Salmonella paratyphi* [160], and in vitro studies have shown its high efficacy against both MSSA and MRSA [131,133]. Tyagi et al. [132] found that one of the antimicrobial activities of curcumin, like other plant-derived antimicrobials, was its ability to damage bacterial cell membranes. This also helps with inhibiting biofilm formation, as curcumin can inhibit bacterial quorum sensing, which in turn prevents bacterial cells from aggregating [130], and it can also inhibit biofilm formation via the production of reactive oxygen species (ROS) [161]. Recent studies have also determined that curcumin inhibited *S. aureus* biofilm formation via downregulation of aggregation and adherence genes, most notably *fnbA* and *clfA*, and interaction with biofilm-forming proteins [134,135,136].

Similarly to the abovementioned antimicrobials, other natural antimicrobials produce synergistic effects when in combination with conventional antibiotics. Palaniappan and Holley [141] demonstrated that pairing individual natural antimicrobials (eugenol, thymol, carvacrol, cinnamaldehyde, allyl isothiocyanate) with antibiotics increased the antibiotics’ effectiveness against resistant bacteria. They found that carvacrol, thymol, and cinnamaldehyde were effective in reducing the resistance of *S. aureus* to various antibiotics including ampicillin, penicillin, and bacitracin. OLE and its major phenolic secondary metabolite, hydroxytyrosol, were found to increase the effect of ampicillin in vitro against *S. aureus* (ATCC 25923), as compared to when ampicillin was used alone [162]. Another study conducted by Mun et al. [131] demonstrated the synergistic effect of curcumin when paired with oxacillin, ampicillin, ciprofloxacin, and norfloxacin, which helped reduce the MICs of these antibiotics against MRSA. Cranberry fractions also showed antibacterial activity against MRSA and MSSA both in vivo and in vitro when used in combination with β-lactams by significantly reducing the MIC (up to 512-fold) of amoxicillin and oxacillin [163].

Taken together, past and recent research highlights the broad-spectrum activity of plant-derived antimicrobials, as they can be used alone or synergistically with other antimicrobials to combat foodborne pathogens, especially *S. aureus*. Plant-derived antimicrobials, including essential oils and extracts, are a promising method of controlling *S. aureus*, including both MRSA and MSSA, due to their wide range of activity, including cell membrane damage, disruption of biofilm formation, and reduction in virulence gene expression. They are recognized as GRAS and are selective in targeting pathogenic bacteria versus beneficial bacteria, and they can enhance the effectiveness of conventional antibiotics, lower required drug concentrations and reduce the risk of resistance. While more research is needed to understand the mechanisms and new synergistic effects, plant-derived antimicrobials offer a safe and alternative strategy to traditional antibiotics.

### 4.3. Antimicrobial Peptides and Nanoparticles

During the last few decades, nanoparticles (NPs) have evolved to be promising antimicrobial agents because of their unique physical and chemical properties and large surface to volume ratios [164,165,166]. Metallic NPs such as silver, copper, titanium, zinc, and their oxides have been reported to have bactericidal properties [167,168]. Silver compounds can easily cross the plasma membrane of a bacterial cell and interact with bacterial genetic information, and this often generates reactive oxygen species (ROS) inside the cell [169]. However, despite their potential as antimicrobials, further research is needed on metallic NPs for their use in the food industry, as studies have shown their potential cytotoxicity [170,171,172].

On the other hand, non-metallic NPs, such as those made from polymers (e.g., chitosan or poly (lactic-co-glycolic acid) (PLGA)) or lipids, have shown promise in inhibiting pathogens, especially *S. aureus*, as they can be engineered to enhance the delivery and efficacy of antibiotics, allowing for improved penetration and accumulation at infection sites and bypassing biofilms [173,174]. Chitosan, derived from chitin, has been used extensively for drug delivery via encapsulation, which allows the release of antibiotics and antimicrobials in a controlled and sustained manner to improve efficacy and reduce side effects [175,176]. Other studies have shown that chitosan also has a direct antimicrobial effect. The positive charge of chitosan allows it to be more strongly absorbed into the surface of a bacterial cell, disrupting the cell membrane and causing leakage of intracellular compounds while also inhibiting the biofilm-forming ability of *S. aureus* [177,178]. While no studies have shown if lipid-based NPs have inherent antimicrobial activity, they are commonly used for encapsulation of antimicrobials due to their non-toxic and biodegradable properties, which results in an improved efficacy in preventing food spoilage [179,180,181,182]. Building on the advances in NP-based delivery systems, antimicrobials, including antimicrobial peptides or plant-based antimicrobials, can be integrated with NPs to further enhance their activity and stability.

**Figure 3:**
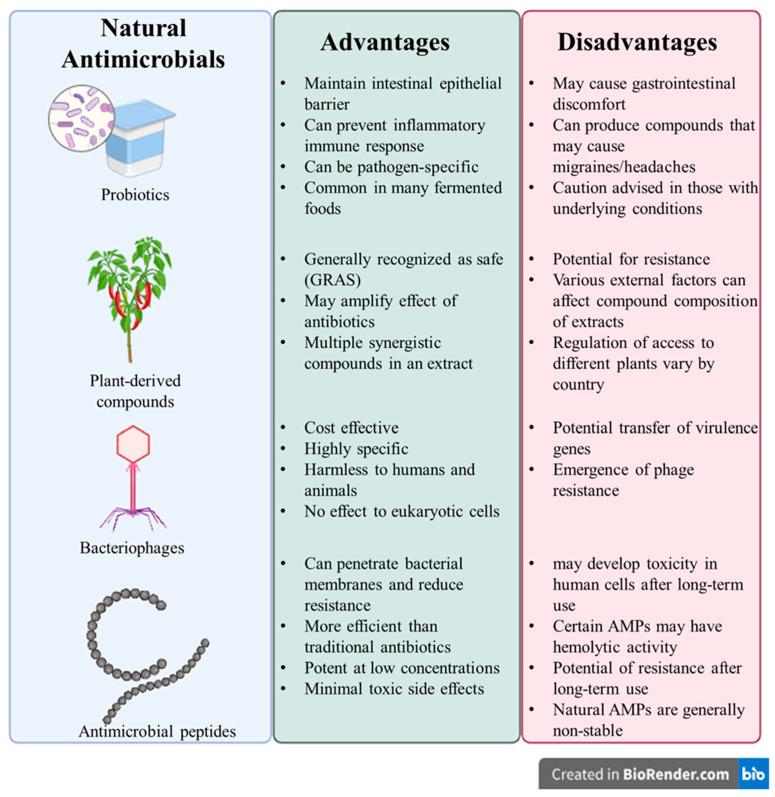
Sources of natural antimicrobials and their advantages and disadvantages in a clinical setting.

Antimicrobial peptides (AMPs) are host defense peptides that are ubiquitous in nature, and they are produced by various organisms such as bacteria, fungi, animals, and plants [183]. AMPs are usually cationic and amphiphilic helical structures, which lead to their activity as antimicrobials. Due to their positive charge, AMPs can bind to negatively charged bacterial cell membranes, which can cause changes in electrochemical potential, induce membrane damage, and allow permeation of larger molecules, leading to leakage of intracellular components and cell death [184]. They have been shown to have a broad-spectrum of antimicrobial activity, with activity against bacteria, fungi, viruses, and even cancers, and they can be used to help overcome drug resistance in bacteria [184]. Using AMPs has shown advantages over traditional antibiotics (Figure 3), and they have been studied as an alternative to chemical food preservatives to prevent the growth of various food spoilage bacteria [185]. Given their potent antimicrobial properties and versatility, recent research has focused on methods for AMP production, particularly the use of microbial fermentation, as a scalable and sustainable approach to meet growing demand for these valuable compounds [186].

Fermentation is a popular method of food preservation, and bacteria such as lactic acid bacteria (LAB) are typically used due to their secretion of AMPs, which can inhibit the growth of pathogenic bacteria in certain food products like dairy products [186]. Nisin is an AMP isolated from bacteria, and it is a type of bacteriocin that is used in various food products. Currently, it is the only AMP that is widely used in the preservation of various food products [187]. Along with the other bacteriocins, such as pediocin and enterocin, nisin has been shown to exhibit antimicrobial activity against the food pathogens often found in dairy products, such *L. monocytogenes*, *E. coli*, and *S. aureus* [188]. Nisin has also shown activity against *Clostridium* and other spore-forming bacteria found in canned foods that survive the elevated temperatures of the canning process [188,189], highlighting the potential of AMPs in food production.

The use of NPs has also recently extended into the field of food science, primarily in extending the shelf life of various food products. AMPs are prone to degradation by proteases and acids in the environment, and some, especially natural AMPs, are not heat-stable. These interactions may decrease the effectiveness of the AMPs, leading to inefficient antimicrobial activity [184,190]. One strategy of overcoming AMP degradation is the encapsulation of natural AMPs by using NPs. NPs have already been used in various food applications, such as flavor stabilization, taste masking, and improvement of shelf life, and their combination with AMPs is a promising method of food preservation [191]. For example, chitosan NPs loaded with nisin decreased *S. aureus* and *L. monocytogenes* populations by 5- to 7-fold when compared to free nisin [192], and AMPs conjugated to PLGA- polyethylene glycol NPs accelerated killing kinetics of the AMPs against *P. aeruginosa* and *S. aureus* [193].

AMPs and NPs provide a promising alternative strategy for controlling *S. aureus*, including antibiotic-resistant strains. AMPs exhibit activity by damaging bacterial cell membranes, and they are able to act against a variety of bacteria, viruses, and fungi. They have been shown to inhibit the growth of many food spoilage bacteria, making them a potentially safer alternative to chemical food preservatives. AMPs have also been used in conjunction with organic-based NPs that provide encapsulation, which allow a more controlled delivery and release of AMPs into the desired environment. Some NPs have also been shown to have antimicrobial effects themselves, damaging bacterial cell membranes and disrupting biofilm formation. Thus, the use of AMPs, NPs, and the encapsulation of AMPs within NPs shows promise as an alternative method of reducing *S. aureus* contamination in food products while also mitigating the risk of the development of antibiotic-resistant strains.

### 4.4. Light-Based Methods

Light-based technologies have become a promising method of controlling bacterial growth, as they are non-thermal and non-chemical-based methods of disinfection. Ultraviolet (UV), blue light, pulsed light, and photodynamic inactivation are methods that have been used in the food industry due to their minimal effect on food quality and nutritional value, and they can be used in combination with other antimicrobial methods at various stages of food production and processing [194]. Light-based technologies, especially UV, are typically used for disinfection of liquid foods and beverages such as milk, juices, and honey, but studies have shown that its application can be extended to the disinfection of produce and meat products [194]. In produce, Kim and Hung [195] demonstrated that treatment with UV-C significantly reduces the growth of *E. coli* O157:H7 on blueberries compared to other sanitizing methods, and Ge et al. [196] showed that treatment of contaminated water with UV-C reduced the concentration of internalized *S. Typhimurium* inside leafy greens.

The use of blue light has also been studied for use in food applications, as it has been shown to have minimal effects on the physical and nutritional quality of food, similar to UV. Blue light is often used in conjunction with photosensitizers, molecules that can be found inside the cell or added externally that can absorb light and become excited to generated reactive oxygen species (ROS), which in turn can be used to target bacterial cells [197]. By itself, blue light has been shown to be an effective antimicrobial, and it is an effective method for sanitizing liquids, especially milk. dos Anjos et al. [198] demonstrated the effective of blue light use on pathogens found in milk, resulting in a reduction of over 5-log of all tested bacterial strains within less than 2 h of treatment while major milk components, excluding riboflavin, had not significant alterations to their chemical structure. While not as effective compared to treatment in liquids, blue light has shown antimicrobial activity when used on solid food products, such as fruits, vegetables, and meats, although it showed varying effectiveness in flat-surfaced foods (cucumbers, tomatoes, etc.) versus those with more complex structures (seeds, strawberries, etc.) [199,200,201].

With light-based technology already being commonly used as an antimicrobial in the food industry, it has also shown promise in controlling the growth of *S. aureus*, which can greatly reduce the risk of contamination of both food products and food processing equipment. Research has shown that UV has been an effective method of inhibiting *S. aureus* growth, particular short-wavelength UV-C. Exposure to UV-C for as little as 10 or 30 min can significantly inhibit the growth of *S. aureus* to almost undetectable levels, and it has also been shown to inhibit biofilm formation by reducing viable growth during biofilm formation by 3-logs [202,203]. Other studies have shown that UV or visible LED can be used in conjunction with other natural antimicrobials, making *S. aureus* more susceptible to their antimicrobial activities. Curcumin has been well studied in inhibiting the growth of *S. aureus* when used with light-based therapies, as it is used as a photosensitizer for photodynamic inactivation. Tortik et al. [204] demonstrated that when blue light was used in combination with curcumin, *S. aureus* growth was decreased by 2.5–2.6-logs on peppers or cucumbers. Other studies have shown that incubating *S. aureus* in the presence of curcumin before exposure to UV-C significantly reduced growth on various food products such as beef, chicken, pork, and fruits in a concentration-dependent manner. Higher concentrations of curcumin increase the effective of UV-C light on *S. aureus* growth, leading to a significant inhibition of 5-logs during biofilm formation when compared to incubation with lower concentrations of curcumin [205,206].

Light-based technologies such as UV or blue light have already been established as a strategy against bacterial pathogens in the food industry, and these methods are valued for their ability to control bacterial growth while also minimizing impact on the physical and nutritional qualities of various food products. By using light-based technologies in combination with other natural antimicrobials or photosensitizers such as curcumin, the contamination of both solid and liquid food products by *S. aureus* can be greatly decreased while also avoiding the risk of antimicrobial resistance. While more research is needed on the application of light with other natural antimicrobials, current trends show that light-based technologies are a promising method for enhancing food quality and safety during both processing and storage.

### 4.5. Vaccines

Vaccination is another useful method of controlling bacterial pathogens, and the use of vaccines in farm animals has been one of the most successful approaches in preventing animal deaths from several bacterial pathogens [207]. Currently, there are four types of vaccines used against bacterial infections: inactivated bacterial pathogens (whole cell antigen) vaccines, live attenuated bacterial vaccines, toxoid and subunit vaccines, and polysaccharide conjugate vaccines. Whole cell antigen vaccines are created by treatment with heating, irradiation, or chemical inactivation of bacteria to attain intact bacteria and their antigens [208]. These vaccines are typically used against extracellular bacteria such as *Vibrio cholerae* [209] but are not very efficient against intracellular bacteria [208]. Live attenuated vaccines are derived from a live infectious agent that has lost its pathogenicity but can induce antibody generation and allow pathogen recognition via the MHC-I presentation pathway [208]. Toxoid vaccines comprise inactivated exotoxins released by bacteria, such as *C. botulinum* and *S. aureus* [208]. Instead of targeting the bacteria themselves, these vaccines generate a neutralizing response against the toxins [210,211]. Polysaccharide conjugate vaccines contain various bacterial cell wall polysaccharides, which provide protection against phagocytosis. To generate an immune response, these vaccines also contain antigenic peptides [208].

In most cases, using antimicrobials against foodborne enteric pathogens may not be an effective strategy because it can lead to the development of antimicrobial resistant microorganisms. Many foodborne pathogens such as *E. coli*, *Salmonella*, *Campylobacter*, and *Shigella* have already developed resistance to several antimicrobials through unregulated utilization of antimicrobial treatment [212]. To overcome the existing problem of antibiotic resistance in foodborne pathogens, vaccine development could be the promising option to succeed in the control of foodborne infections [213]. Despite the success of vaccinations against many viruses and several bacteria, some difficulties in vaccination against bacteria are still impeding progress. Bacteria are more complex than viruses and have a variety of antigens, and it is still unclear which antigens can trigger a protective and long-lasting immune response. Many past and current bacterial vaccines have been successfully developed against extracellular bacteria, but intracellular bacteria still pose an issue due to their ability to naturally evade most immune responses [208].

Currently, there are no approved vaccines for *S. aureus*. This is most likely due to a lack of successful translation of vaccine protection observed in preclinical trials to subject subjects [214]. Previous vaccine candidates, such as the StaphVAX and V710, which target individual cell surface components, showed efficacy in preclinical animal models but failed to show efficacy in Phase III trials [215,216]. Failure of these vaccine candidates most likely result from the presence of multiple virulence factors, such as Protein A, which interacts with the Fc portion of immunoglobulin, limiting B-cell antibody production [217]. Due to the need for a vaccine with multiple antigen preparation, Moscoso et al. [218] developed a live-attenuated vaccine candidate that was shown to be protective in a mouse model. Their findings demonstrated that the vaccine elicit had a protective immune response that also produced cross-reactive antibodies, resulting in decreased bacterial loads. However, it still needs to be tested in other animal models before proceeding to human clinical trials.

More recent vaccine studies have elected to investigate toxoid vaccines, which aim to target and neutralize staphylococcal toxins. IBT-V02, currently in Phase I trials, is a multivalent toxoid vaccine comprising 6 weakened toxins, including α-hemolysin, Paton-Valentine leucocidin, leucocidin AB, toxic shock syndrome toxin-1, and staphylococcal enterotoxins A and B. It was developed with the aim of treating skin and soft-tissue infections, and murine and rabbit models showed that it induced the production of toxin-neutralizing antibodies with a protective efficacy against MRSA and multiple clinical isolates [219]. Poolman et al. [220] developed the SpA + LukAB vaccine, comprising an inactivated SpA protein and LukAB toxoid, to target immune evasion factors. Using a minipig model, they demonstrated that SpA + LukAB was able to cause a significant reduction in bacterial load and dissemination in surgical site infections and decreased severity in wound infection. Other vaccine candidates are in various stages of development, such as whole-cell vaccines and recombinant antigen-based vaccines, though none have yet to be approved for human use [221,222].

### 4.6. Limitations of Above-Mentioned Interventions to Control Foodborne Diseases

Despite several promising interventions to control foodborne pathogens, foodborne bacterial pathogens continue to jeopardize the health and lives of millions of people worldwide each year. The challenges of the existing interventions, such as antibiotics, are directed towards the emergence of antimicrobial resistance in bacteria. This can make these bacteria nearly impossible to entirely control [213]. However, natural antimicrobials also face their own challenges when it comes to bacterial resistance as well as development (Figure 3).

While phages can be an effective treatment against bacterial pathogens, there are several limitations. Recent studies have shown that phages are able to enter human cells, but there is little evidence on how they interact with intracellular pathogens [223,224]. Another limitation of phages is their ability to facilitate horizontal gene transfer [225]. Transferred genes may carry antibiotic resistance and can increase the virulence of a pathogen [226], which may lead to the evolution of more antibiotic-resistant bacteria [225,227]. Moreover, pathogens can develop several resistance mechanisms against phages, involving the modification or loss of receptors for phage attachment, secretion of molecules to prevent phage adhesion, prevention of phage DNA injection into the bacteria, inhibition of the release of phage DNA and multiplication, and abortive infection [228]. The possibility of bacterial resistance to phages has been demonstrated by several researchers using *E. coli* [229], *S. aureus* [230], *Salmonella* [231], and *V. cholerae* [232] through the change or loss of receptors by membrane protein modifications and abortive infections. Nonetheless, phage therapy is still a strong candidate for alternative treatment due to a relatively narrow host range, which can only affect few bacterial strains or species of interest [98,233].

Natural antimicrobials can be a safer alternative to using only synthetic antimicrobials, and their applications in food safety have already been well-studied. However, although many plant-derived antimicrobials have been shown to prove effective against various pathogens, there are still challenges to overcome, including variability of plant compounds, and unintended side effects [234]. Antimicrobial susceptibility tests have shown variability in the antimicrobial activities of plant extracts, indicating a lack of standardization during the growth and/or extraction process of the plants. This variability may be due to multiple factors, including differences among plant species, growing conditions, extraction methods, and compound concentrations [235]. Plant-derived antimicrobials have been frequently reported to be less toxic, but they may cause cytotoxic effects if the proper dosage is not used, a potential outcome from the lack of standardization. For example, Kengni et al. [236] showed that *Harungana madagascariensis* leaf extract had strong antibacterial activity against *S.* Typhimurium, but higher doses could induce liver damage and increased cholesterol in mice. In addition, many other plant extracts have not been evaluated by the FDA, and there is a lack of comprehensive data on their toxicity [235]. While plant-derived antimicrobials are a strong contender in the fight against multidrug-resistant bacteria, the lack of comprehensive knowledge and standardization is a serious limitation against their clinical use.

As mentioned above, the lack of an approved vaccine against *S. aureus* is a major limiting factor in the use of vaccines against SFP. Several vaccines have been developed to attack cell surface components, such as polysaccharide molecules, or cell wall proteins that help in adhesion, invasion, or reception. However, these vaccine candidates only seem promising in earlier clinical phase development in animal models but have failed to show efficacy in human trials [237]. *S. aureus* evades host immunity through the polymorphic expression of surface antigens and release of several virulence factors [224,225], including iron acquisition factors such as IsdB, fibronectin binding proteins, polysaccharide capsule molecules, and toxins [238]. These evasion strategies suggest that vaccination against *S. aureus* may be challenging, and more research and trials need to be performed to translate preclinical results to human trials.

The use of light-based treatments, especially UV, has already been well-documented in the food industry, as some companies have already implemented their use in sanitizing food and water. Although lauded for their minimal effect on food quality and lack of residue, light-based treatments still have limitations. UV and other similar treatments have limited penetration power, as they primarily act on exposed surfaces and cannot penetrate through most food items. Foods with an irregular or porous shape, such as berries or meat, can harbor bacteria within their crevices or below their surface, shielding them from light exposure [194,239]. Adhikari et al. [240] demonstrated that UV-C treatment of apples and pears resulted in a 2.1–2.9-log reduction of *E. coli* O157:H7, but treatment of strawberries and raspberries, whose surfaces are pitted compared to the smooth surface of apples or pears, at a higher intensity resulted in only 1.1–2-log reduction. A similar study also showed that the use of UV on intact pear surface compared to bruised pear surface resulted in a 0.8-log difference in reduction of *E. coli*, with the intact pear surface having a greater reduction [241]. Light-based have also been shown to have no standardized dosing, with some methods requiring longer exposure times and other methods having uneven dosing, which may lead to inconsistent treatment preventing the complete eradication of food spoilage bacteria [204]. While light-based treatments are a promising method of controlling bacterial growth in food due to their minimal effect to the food products, their effectiveness remains inconsistent among different food types, preventing standardized protocols.

There are several limitations on the use of NPs as an intervention mechanism. Most studies conducted have so far been focused on *E. coli* strains; the bacteria and NP-based intervention strategies in bacterial pathogens is only beginning to occur in *L. monocytogenes*, *Staphylococcus*, *Salmonella*, and *P. aeruginosa* in the context of intracellular infections [242]. A major concern of NP use is their potential toxicity to human cells. As previously stated, although their antimicrobial effects are widely noted, metallic NPs, such as silver, have been reported to be cytotoxic, raising concerns about their clinical applications [243,244,245]. On the other hand, although non-metallic NPs, such as those based on polymers or lipids, offer a potentially safer method, they face several limitations. NP effectiveness is highly dependent on particle size, structure, and surface properties, which adds difficulty in production and standardization [246]. Additionally, the mechanism of their antimicrobial effects is not well understood, as different formulations with other antimicrobials may yield inconsistent results [247]. More research is needed to evaluate the mechanisms and safety of nanoparticle usage, especially in food safety, before they can be readily used for consumers.

**Figure 4:**
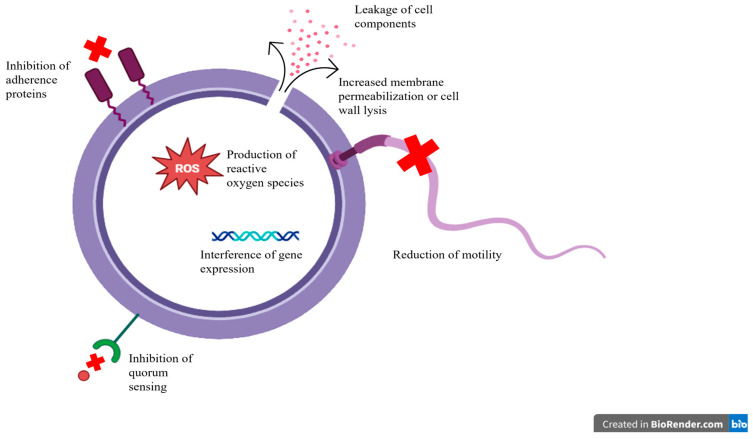
The mechanisms of action of plant-based antimicrobials or antimicrobial peptides. Antimicrobial activity results in inhibition of adherence proteins, inhibition of quorum sensing receptors, damage or lysis of cell membrane or wall, reduction in motility, production of ROS, and/or interference of metabolic or virulence gene expression.

The growing threat of antibiotic resistance among foodborne pathogens, particularly *S. aureus*, has spurred the development and evaluation of alternative antimicrobial strategies, each with distinct mechanisms and advantages. While these alternative strategies provide a diverse array of methods to control the growth of food spoilage bacteria, especially *S. aureus*, the ubiquitous nature of *S. aureus* and the inherent limitations of the described techniques prevents one method from being the priority choice of treatment. Phage therapy leverages the species- and strain-specificity of phages to selectively target pathogenic bacteria while not harming beneficial bacteria [100,101,102]. Numerous studies have shown that lytic phages and phage-derived endolysins have potent, targeted antibacterial effects against *S. aureus*, including MRSA, in both animal trials and food industry settings [107,108,109,112,113]. This high specificity, safety profile, and lack of eukaryotic toxicity make phages attractive, though their narrow host range may limit coverage and the risk of horizontal gene transfer from temperate phages must be carefully managed [90,225]. In contrast, plant-derived antimicrobials such as essential oils (EOs), olive leaf extracts, and curcumin, offer broad-spectrum antibacterial effects by compromising microbial cell membranes and inhibiting biofilm formation (Figure 4) [119,121,126,143]. These natural substances are generally recognized as safe (GRAS), readily accessible, and have shown selective activity against pathogens over commensals [118], and their wide applicability and multiple biological targets reduce the likelihood of resistance. However, variability in their effectiveness, lack of standardization of dosages, and limited stability under processing conditions must be considered [235,236].

Similarly to plant-based antimicrobials, AMPs mainly disrupt bacterial membranes while also displaying broad-spectrum activity, including against resistant strains (Figure 4) [184]. Most non-metallic NPs are typically used to deliver AMPs or traditional antibiotics in a targeted, sustained manner, and some, such as chitosan NPs, possess intrinsic antibacterial properties [173,176]. The combination of AMPs with NPs addresses issues of stability and delivery in complex food matrices, but despite their promise, concerns about cytotoxicity, especially with metallic NPs, remain a major factor in preventing AMPs and NPs from being readily used by consumers [246,247]. Light-based technologies, on the other hand, offer non-chemical and non-thermal approaches to controlling pathogens on food surfaces and in processing environments. Unlike AMPs and NPs, they are residue-free and are not consumed by consumers, alleviating the risk of cytotoxicity of treated food products [194,197]. However, like with the previously described antimicrobial strategies, it is difficult to standardize the dosage of various light-based treatment, providing the same challenge in providing a common protocol for companies throughout the food industry [194,198,200]. Therefore, researchers are also focusing on the gut microbiome and their modulation with probiotics, prebiotics, or their combination in stimulating host immunity and competitively excluding colonization of pathogens, known as “pro-commensal strategies”.

## 5. Advantages of Pro-Commensal Strategies in Control of Foodborne *S. aureus*

### 5.1. Probiotics

Probiotics are microorganisms, such as *Lactobacillus* and *Bifidobacterium*, which are non-pathogenic and aid in balancing the intestinal epithelium and defending against infectious agents. Probiotics are crucial in maintaining intestinal homeostasis, as disruption of the gut microbiome has been correlated with issues in the cardiovascular system, gastrointestinal tract, liver, and mental health [248]. Probiotics are often naturally found or added to fermented foods, and they can be administered as dietary supplements [249]. Certain characteristics of probiotics help them colonize and survive on the mucosal surfaces of the gut. Several studies found that probiotics can prevent colonization of enteric pathogens by maintaining the intestinal epithelial barrier, activating, and modulating innate and acquired immunity, and/or producing organic acids, hydrogen peroxide, bacteriocins and other antimicrobial compounds [250,251]. The interaction between probiotics and the immune system is fostered by both the prevention of inflammatory responses and the enhancement of the immune system. This mechanism of immune response is facilitated through the inhibition of NF-KB pathways and the promotion of pro-and anti-inflammatory cytokine secretion [252].

Lactic acid bacteria (LAB) are Gram-positive bacteria that produce lactic acid as a fermentation product. They are commonly used as starter cultures in fermented foods and dairy products, and most are considered GRAS. LAB can secrete antimicrobial substances, such as bacteriocins, that exhibit bacteriostatic or bactericidal properties that inhibit the multiplication of pathogens. There are several diverse types of bacteriocins produced by LAB isolated from different foods, and they provide an antagonistic environment against many pathogenic bacteria such as *Listeria*, *Clostridium*, *Staphylococcus*, and *Bacillus* [253]. The efficacy of probiotics has been evaluated in the control of several foodborne pathogens such as *Shigella sonnei*, *E. faecalis*, *Proteus mirabilis*, *P. aeruginosa*, and *S. aureus* [254,255]. *Lactobacilli* and *Bifidobacteria* have been found to increase colonization resistance and inhibition against *H. pylori* [256,257], *E. coli* 0157:H7 [258], and *Salmonella* [259] in vivo and in vitro, demonstrating their importance in the human gut.

Probiotics in the gut create an environment for competition by exclusion, where enteric pathogens are forced to compete for adhesion sites or nutrients. Several pathogenic bacteria, such as *L. monocytogenes*, *Salmonella*, *E. coli*, and *Clostridium difficile*, have been reported to have reduced intestinal colonization with probiotic intervention [260,261]. The interaction between probiotics and host gut epithelium results in a greater production of mucus, which strengthens the intestinal physical barrier against foodborne enteric pathogens [262]. Probiotics are also known to modulate cytoskeleton structures in the intestinal mucosa and exhibit inhibitory effects on pathogenic bacteria [263]. Fang et al. [264] investigated the inhibitory effects of *Lactobacillus casei* subsp. *rhamnosus* (Lcr35) on the anti-inflammatory effects and reinforcement of the intestinal epithelial barrier against *Salmonella* toxins. Other *Lactobacillus*, such as *L. fermentum*, *L. plantarum*, and *L. paracasei*, also have inhibitory effects against entero-pathogenic *E. coli* (EPEC) due to the production of organic acids or hydrogen peroxide [265]. Due to their documented activity against other pathogens, probiotics, especially LAB, are a promising alternative for controlling *S. aureus*.

The use of probiotics against *S. aureus* has been well documented (Table 2). Clinical trials have shown that oral administration of *Bacillus subtilis* can significantly reduce *S. aureus* colonization in human gut and nasal passages. Specifically, *B. subtilis* secretes fengycins, a type of lipopeptide, that disrupts *S. aureus* quorum-sensing, which leads to disruption in biofilm formation and colonization [266]. Other studies highlight the strong antimicrobial activity of LAB against *S. aureus*, such as *L. rhamnosus* and *Lactobacillus acidophilus*, through the use of secreted antimicrobials, modulating host immune system, and competition for nutrients and binding sites [267,268]. Administration of LAB has been shown to decrease *S. aureus* colonization in animal and human models by decreasing the number of *S. aureus* in both the gut and nasal passages, resulting in a more balance microbiome [269,270,271], highlighting the use of probiotics as an alternative strategy to treat *S. aureus* infections while reducing the risk of dysbiosis associated with antibiotics use.

To further study the mechanism of action of probiotics against pathogens, studies have been conducted to better understand these interactions. Tejero-Sariñena et al. [286] investigated the antimicrobial properties of probiotics *Lactobacillus*, *Bifidobacterium*, *Lactococcus*, *Streptococcus* and *Bacillus* against Gram-positive and Gram-negative bacteria. The active compounds from the probiotics showed antagonistic properties against *S. Typhimurium*, *E. coli*, *E. faecalis*, *S. aureus* and *C. difficile*, and they hypothesized that the possible inhibitory mechanism could be due to the production of metabolites, such as organic acids from glucose fermentation, which subsequently lowered the pH of the culture media. Evivie et al. [287] showed that *Streptococcus thermophilus* KLDS 3.1003 has both genes for bacteriocin production and can produce substantial amounts of lactic and acetic acid, which can inhibit *E. coli* and *S. aureus* in vitro and in vivo.

These secreted metabolites additionally contribute to the broad-spectrum activity exhibited by some of these probiotics. For example, in a study conducted by Singh et al. [288], the supernatant of *Lactobacilli* cultures that were isolated from breast milk were evaluated for antibacterial activity against *P. aeruginosa* and *S. aureus*. They found that several strains exhibited antimicrobial and antibiofilm activities against both *P. aeruginosa* and *S. aureus*, which was attributed to the production of organic acids and bacteriocins. In another study, *Lactobacillus crispatus* M247 was shown to have strong, broad-spectrum antimicrobial activity in an in vitro environment against a range of common vaginal dysbiosis pathogens, including, but not limited to, *S. aureus*, *E. coli*, and *E. faecalis* [289]. Sikorska and Smoragiewicz [290] also demonstrated that *L. acidophilus* CL1285 and *L. casei* LBC80R were effective against clinical MRSA isolates and *S. aureus* biofilm formation. Additionally, they found that *L. plantarum* showed the highest inhibitory effect against *S. aureus* in vitro, and it was also very effective in controlling skin wounds infected with *S. aureus* in mice when used topically. Overall, many have described these inhibitory effects of probiotics as being mediated through direct cell competitive exclusion and production of acids or bacteriocin-like inhibitors.

Several probiotics also produce biosurfactants, which are amphipathic compounds that have antibacterial properties. The inhibition of bacterial growth and biofilm formation properties via biosurfactants produced by *Lactobacillus* was presumed to be due to the altered hydrophobicity of the bacterial cell surface [291]. The biosurfactants produced by *L. helveticus* were shown to inhibit biofilm formation both in vivo and in vitro, as well as demonstrating anti-adhesive properties against *S. aureus* [292]. Biosurfactants from *L. paracasei* subsp. *paracasei* A20 have also been reported to exhibit anti-adhesive and antimicrobial properties against several pathogens, such as *E. coli* and *S. aureus* [293]. Biosurfactants isolated from *Pediococcus acidilactici* and *L. plantarum* exhibit anti-adhesive and anti-biofilm properties against *S. aureus* through the suppression of biofilm-related genes and interference with the release of signaling molecules [294].

Although probiotics provide numerous benefits to the body, some individuals may experience side effects from the readjustment of the microbiome towards the addition of probiotics. Some have reported mild gastrointestinal symptoms such as bloating, gas, diarrhea, and constipation, though symptoms normally resolve within a few weeks [295]. As they are live microorganisms, caution must be taken in those with underlying health conditions, such as immunodeficiency, acute abdominal or intestinal infection, or heart disease, to prevent potential infection [296]. Additionally, the efficacy of the probiotics is also dependent on the probiotic strain, dose, route of administration, and the probiotic formulation [297], so care must be taken into account when addressing individual patients.

### 5.2. Synbiotics

The Food and Agriculture Organization (FAO) and WHO have defined prebiotics as non-digestible products that selectively stimulate the growth or activity of a certain number of indigenous bacteria in the host. The favorable changes created by prebiotics in microbiota carry significant importance for maintaining and strengthening the host health. Prebiotics are primarily part of oligosaccharides and are abundantly found in fruits, cereals, and vegetables, such as beet, garlic, onion, honey, banana, wheat, barley, tomato, and bean [298]. Prebiotics can survive the enzymatic action in the stomach and are processed by gut microbiota into beneficial short chain fatty acids upon reaching the large intestine [299]. These prebiotics can be used in combination with probiotics to maximize the efficiency of both, forming what are called synbiotics.

The combined actions of prebiotics in the large intestine and of probiotics in small intestine provide tremendous synergistic benefits to the host’s health, such as improvement of immune function [300]. For example, Li et al. [301] found that symbiotic supplementation of *Bifidobacterium lactis* or *L. rhamnosus* with fructooligosaccharide had greater reductions in plasma C-reactive protein compared with a placebo group. Supplementation with synbiotics also resulted in an increase in beneficial bacteria, which also correlated with an increase in amino acid and SCFA biosynthesis pathways. The combination of fructooligosaccharide with *Lactobacillus* and *Bifidobacterium* was also shown to decrease the production of pro-inflammatory cytokines while the expression of anti-inflammatory cytokines was increased in rats, suggesting that synbiotics are a lucrative method of modulating the gut microbiota to improve immune function [302]. Kariyawasam et al. [303] demonstrated that *Lactobacillus brevis* supplemented with fructooligosaccharides exhibited improved viability compared to non-supplemented controls, and supplemented *L. brevis* also exhibited higher antimicrobial activity against multiple foodborne pathogens, including *L. monocytogenes*, *S. aureus*, *E. coli*, and *S.* Enteritidis. Prebiotics have also been shown to increase probiotic concentrations in the intestinal tract, an example of which is soy insoluble dietary fiber providing an ideal surface for adherence, which aids in probiotic proliferation [304,305]. Recent studies highlight the potential of synbiotics as a method of food preservation. Advances in encapsulation have also been shown to improve the use of synbiotics, as demonstrated by Malos et al. [306]. They found that encapsulated synbiotics, specifically *L. plantarum* with fructooligosaccharides and inulin, improve probiotic viability under simulated gastrointestinal conditions. Other studies showed that integrating probiotics and prebiotics simultaneously in both dairy and non-dairy products increased the effectiveness of both compounds, supporting gut health and improved nutrient absorption [307,308,309,310]. Although synbiotics have shown to be a promising method to control bacterial infections and food preservation, very few have been shown to have clinical efficacy [311]. Ongoing research and technological advances are needed to better understand and optimize their use in food systems.

## 6. Conclusions

Increasingly, the prevalence of SFP and its impacts on consumer health are issues in the modern world. This review indicates the increasing trends of SFP in both developing and developed countries. To date, various interventions have been developed to inhibit *S. aureus* and MRSA both in vitro and in vivo. However, challenges in cost effectiveness, environmental friendliness, antimicrobial resistance, and strain specificity still exist. We summarize the possibility of using bacteriophages, plant-derived antimicrobials, NPs, probiotics, and synbiotics to effectively reduce SFP as an alternative to existing control strategies. Phages offer targeted antimicrobial activity against various *S. aureus* strains, while plant-based antimicrobials provide broad-spectrum inhibition with minimal environmental risks. NPs can be engineered for enhanced delivery and sustained antimicrobial effects, and probiotics and synbiotics contribute to pathogen reduction by competitive exclusion, immune modulation, and the production of antimicrobial substances. Light-based treatments provide an effective method of surface-level reduction of *S. aureus*, and it has been found to increase in efficacy when used in combination with other antimicrobial methods. However, numerous factors, such as the selection of strains and doses, the route of administration, and the formulation of antimicrobial preparation, need to be considered for effective inhibition of foodborne pathogens. Therefore, future research should focus on advancing knowledge of natural antimicrobial mechanisms as well as the combination of multiple strategies, such as synbiotics with nanoparticles or antimicrobials with light-based technologies, to increase the understanding of their mechanisms of action with each other and against *S. aureus*. Overall, studies should aim to enhance the stability, delivery, and synergistic potential of these strategies in different food systems while also minimizing disruption of beneficial bacteria as well as food quality. Such research will aid in establishing these methods as a successful approach to overcome prevailing food poisoning problems in the world.

## Figures and Tables

**Table 1:** Major plant-derived antimicrobials and their mode of action.

Natural Antimicrobial Sources	Mechanism of Action	References
Citrus oil	-downregulation of cell wall synthesis genes -Induction of cell wall damage and lysis	[119,120,121,122,123,124,125]
Olive leaf extract	-reduction of flagella/motility -induction of cell wall and cell membrane damage	[126,127,128,129]
Curcumin	-inhibition of bacterial quorum sensing -inhibition of biofilm formation	[130,131,132,133,134,135,136]
*Inula graveolens* and *Santolina corsica* essential oil	-alteration of cell membrane -induction of cytoplasm leakage	[137]
Thyme essential oil	-damage of membrane integrity -increased membrane permeability -production of ROS -reduction of motility	[138,139,140,141]

**Table 2:** The antagonistic activity of probiotics against food poisoning *S. aureus*.

Probiotic	*S. aureus* Strain	Antagonistic Activity	Mechanism	Reference
*L. fermentum RC-14*	*S. aureus* ID_50_ (Oxford strain)	Inhibited *S. aureus* infection of surgical implants, completed inhibited subcutaneous abscess formation	Inhibition of adhesion to surfaces in vitro	[272]
*L. plantarum* MH734175, *Leuconostoc mesenteroides* CAU111	*S. aureus* subsp. *aureus* PTCC 1431	Antagonistic and proteolytic activity of probiotics against *S. aureus* with a zone of inhibition of 0.5 mm or larger	Bactericidal effect of protease-sensitive bacteriocins	[273]
*L. acidophilus* NK1, *Bifidobacterium adolescentis* MC 42	Clinical strains of *S. aureus* (478, 502, 46 m, 83p)	Destructive properties against *S. aureus* biofilm	Biofilm degradation, biofilm refinement, segmentation, detachment of fragment and lysis	[274]
*L. reuteri L 22*	*S. aureus* ATCC 25923	The numbers of MRSA 02 were significantly lowered by more than 2-log-fold and no growth after 24 h of incubation	Antibacterial properties attributed by the production of H_2_O_2_ and bacteriocin	[275]
*L. acidophilus* CL1285, *L. casei*	*S. aureus* ATCC 29213	The consumption of the CL1285 in mice reduced *S. aureus* after 18 days by 85%	Production of organic acids and bacteriocin	[276]
*L. acidophilus* CL1285 and *L. casei* LBC80R	*S. aureus* ATCC 43300 and MRSA clinical isolate	The inhibition of MRSA growth observed with the inhibition zones ranging from 1.4 to 2.9 cm		[277]
*L. acidophilus* (LA5), *L. casei* 01	*S. aureus* ATCC 29213	The reduction of *S. aureus* up to 3-log/CFU in a co-culture method and suppression of SEA, SEC and SEE production up to 10.31-fold in co-incubation	Production of bacteriocin and hydrogen peroxide, competition for nutrients, and acidification	[278]
*Saccharomyces cerevisiae* S3	*S. aureus* ATCC 29213, *S. aureus* ATCC 33591	Supernatant and lysate extract reduced biofilm formation up to 69% and 80%, respectively. The hemolytic activity was reduced up to 93% by supernatant extract. The reduction of sea gene expression by 12-fold	Mannoproteins extracted *S. cerevisiae* cell wall believed to reduce biofilm growth	[279]
*L. rhamnosus* SHA113	*S. aureus* ZBQ006, *S. aureus* 29213	Up to 79% inhibition of *S. aureus* growth and reduced biofilm formation	Production of organic acids, hydrogen peroxide and biosurfactants. Inhibition of the expression of TNF-alpha and IL-6 Expression of up regulatory tight junction proteins ZO-1 and occluding. inflammatory factors	[280]
*Streptococcus salivarius* K12	*S. aureus* ATCC 25923	Inhibited the formation and maturation of fresh *S. aureus* biofilm by more than 60%	Acidification of the growth media	[281]
*B. subtilis* BSB3, 16k	*S. aureus* 10292, *S. aureus* 10378, *S. aureus* 12600, *S. aureus* 10203, MRSA 13, 34, 26, 2, 5	The inhibition of *S. aureus* observed with zones of inhibition measured up to 20 mm	The production of biosurfactants	[282]
*L. acidophilus* La-5 and *Bifidobacterium longum* ATCC 15707	*S. aureus*	Inhibited the growth with inhibition zone of >17 mm based on agar spot and well diffusion assay	Production of organic acids (lactic acid, acetic acid), hydrogen peroxide and bacteriocins	[283]
*L. acidophilus* AC, *Bifidobacterium bifidum 4*	*S. aureus* ATCC 25903	Inhibited *S. aureus* concentration up to 10^6^ CFU/cm^3^ during joint cultivation at 60 and 72 h of incubation	Competition for nutrients, production of short-chain acids, and inhibitory substances of protein and non-protein nature	[284]
*S. thermophilus* (XN-S)	*S. aureus* ATCC 29213	Cell-free culture supernatant from selenium (Se)-enriched *S. thermophilus* exerted stronger antibacterial activity than those from the non-Se strains	Cell structure damage	[285]

## Data Availability

No new data were created or analyzed in this study. Data sharing is not applicable to this article.
